# Crowdsourcing the nodulation gene network discovery environment

**DOI:** 10.1186/s12859-016-1089-3

**Published:** 2016-05-26

**Authors:** Yupeng Li, Scott A. Jackson

**Affiliations:** Center for Applied Genetic Technologies, University of Georgia, 111 Riverbend Road, Athens, 30602 GA USA; Institute of Plant Breeding, Genetics and Genomics, University of Georgia, 111 Riverbend Road, Athens, 30602 GA USA

**Keywords:** Gene network, Crowdsourcing, Nodule symbiosis

## Abstract

**Background:**

The Legumes (Fabaceae) are an economically and ecologically important group of plant species with the conspicuous capacity for symbiotic nitrogen fixation in root nodules, specialized plant organs containing symbiotic microbes. With the aim of understanding the underlying molecular mechanisms leading to nodulation, many efforts are underway to identify nodulation-related genes and determine how these genes interact with each other. In order to accurately and efficiently reconstruct nodulation gene network, a crowdsourcing platform, CrowdNodNet, was created.

**Results:**

The platform implements the jQuery and vis.js JavaScript libraries, so that users are able to interactively visualize and edit the gene network, and easily access the information about the network, e.g. gene lists, gene interactions and gene functional annotations. In addition, all the gene information is written on MediaWiki pages, enabling users to edit and contribute to the network curation.

**Conclusions:**

Utilizing the continuously updated, collaboratively written, and community-reviewed Wikipedia model, the platform could, in a short time, become a comprehensive knowledge base of nodulation-related pathways. The platform could also be used for other biological processes, and thus has great potential for integrating and advancing our understanding of the functional genomics and systems biology of any process for any species. The platform is available at http://crowd.bioops.info/, and the source code can be openly accessed at https://github.com/bioops/crowdnodnet under MIT License.

## Background

Symbiosis between legumes and rhizobia leads to the development of specialized root organs, called nodules, in which nitrogen-fixing bacteria are accommodated intracellularly and are able to efficiently convert atmospheric nitrogen into ammonia and transfer it to the host plants [1]. The legume nodule symbiosis is a one of the most efficient systems for plants to acquire nitrogen from the atmosphere. Legumes (Fabaceae or Leguminosae) have the unique ability to carry out symbiotic nitrogen fixation with rhizobial bacteria in root nodules. Many efforts are underway to identify nodulation-related genes and their functions in *Lotus japonicas, Medicago truncatula, and Glycine max*, long-established models for the study of legume biology [2–10]. However, owing to the complexity of the transcriptional regulation of root nodule symbiosis, not all nodulation-related genes have been identified. More importantly, the functions of nodulation-related genes and how they interact with each other are even less well understood. Thus, it is necessary to study nodulation using gene network analysis that can help to reveal many, or even most, of the underlying gene interactions and functions [11].

Gene networks or pathways can be computationally predicted using biological data, e.g. trancriptomes, protein-protein interactions, Gene Ontology (GO) similarities and text mining [11]. These computational methods often suffer from high rates of false discovery. For example, very few systematic or genome-wide studies of nodulation gene interactions, other than co-expression, have been done [5, 12, 13]. It is difficult to accurately identify the direct interactions using gene co-expression [14]. Second, most of the nodulation genes have orthologs in other plants, including *Arabidopsis thaliana* [15], and GO annotations of legume genes are often based on the GO terms of their orthologs in *A. thaliana*. Since nodulation is peculiar to legumes and a very few other species, the function of many nodulation genes may differ from their orthologs in non-nodulating species such as Arabidopsis. Therefore, the use of GO annotations from orthologous genes may be misleading. Third, text mining can automatically search the scientific literature and extract information for potential gene interactions. Many tools have been developed to reconstruct gene networks from text mining [16–24]. However, the complexity of natural language and the inconsistent use of gene names make text mining error-prone [25].

We would like to create a user-curated, highly accurate database that would serve as a knowledge base for experimentally verified nodulation genes and their interactions. Furthermore, this resource can also be used as highly accurate prior information in order to increase the power of gene network predictions [26]. Manual curation from peer-reviewed literature is the most accurate, but is time-consuming. In order to increase the efficiency of manual curation, we propose an alternative solution for creating a nodulation gene association database: crowdsourcing. Crowdsourcing is an online activity in which an undefined, but generally large, group of people voluntarily undertake a task via an open call [27]. The most extraordinary example of crowdsourcing is probably Wikipedia (https://www.wikipedia.org/), an online encyclopedia that anyone can edit. The number of Wikipedia articles quickly increased to over four million in the first five years since it was established, and the quality of articles in science was close to those in Encyclopaedia Britannica [28]. The continuously updated, collaboratively written, and community-reviewed wiki model has been applied for both biology and bioinformatics. For example, WikiGenes is a wiki-based platform for the scientific community to collect, communicate and evaluate knowledge about genes, chemicals, diseases and other biomedical concepts [29]. Gene Wiki [30] and LncRNAWiki [31] are similar platforms, but focused on human genes and long non-coding RNAs, respectively. WikiPathways is a wiki-based pathway curation resource, coupled with an graphical pathway editing tool [32].

We present here an online platform for crowdsourcing nodulation gene network reconstruction with three goals: to i) comprehensively integrate knowledge of all known nodulation-related genes and gene interactions in legumes, ii) provide a user-friendly tool for interactively visualizing gene interactions and gene annotations, and iii) utilize the wiki model for collaborative editing.

## Implementation

The platform is open access and open source under MIT License (https://opensource.org/licenses/MIT). The web server is a Ubuntu 14.04.1 virtual machine image pre-built by Bitnami (https://aws.amazon.com/marketplace/pp/B0062NF3ME), and is hosted on a t1.micro instance on Amazon cloud. The image (version 5.5.25-0) has pre-installed software packages for basic web development, including Apache 2.4.12, MySQL 5.6.23 and PHP 5.5.25. The platform has been tested on Firefox 38, Chrome 43, Safari 8, and all features should work in these browsers with equal or higher versions. The platform was written in JavaScipt and PHP, and the source code is hosted on GitHub (https://github.com/bioops/crowdnodnet). With only a few configurations, the source code can be used to build similar platforms for other biological processes. Researchers are welcome and encouraged to modify and improve the source code via GitHub pull requests.

## Results and discussion

### Visualization

**Fig. 1:**
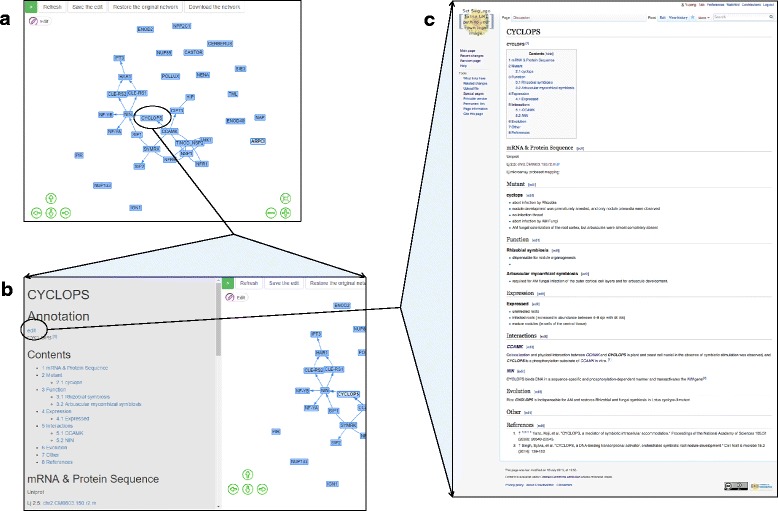
Screenshots of CrowdNodNet. **a** The main page, displaying the nodulation gene network in *Lotus japonicas*. **b** Once a gene (CYCLOPS) is selected, the gene and its edges are highlighted and the gene’s annotation document appears. **c** The MediaWiki page of a gene (CYCLOPS)

The interface of the platform is a web page (http://crowd.bioops.info/) that displays the *L. japonicas* nodulation gene network using vis.js (http://visjs.org/), a JavaScript library for dynamic and interactive data visualization (Fig. 1). The gene network consists of a list of known nodulation-related genes and interactions between these genes, that were manually retrieved from literature. For example, the symbiosis receptor-like kinase (SYMRK) gene, required for nitrogen-fixing root nodule symbiosis of legumes, participates in a symbiotic signal transduction pathway during nodulation [2]. Thus, a node labeled ‘SYMRK’ is shown in the network. Directed and unweighted edges represent gene interactions, e.g. receptor-binding, phosphorylation reactions and protein complexes. For example, CYCLOPS is phosphorylated by the calcium/calmodulin-dependent kinase (CCAMK), and the phosphorylated CYCLOPS becomes an active transcription factor that transactivates the nodule inception (NIN) gene [7]. These interactions are represented by two solid directed edges that point from CCAMK to CYCLOPS, and from CYCLOPS to NIN, respectively. The lists of nodulation related genes and gene interactions currently included in the gene network can be found at http://Crowd.bioops.info/mediawiki/index.php/Nodes and http://Crowd.bioops.info/mediawiki/index.php/Edges, respectively. The gene network can also be downloaded as a json format file.

Users can hover over a gene to see the gene’s full name, and the gene and its edges are highlighted. Users can further click on the gene and a document containing the gene’s annotation information, retrieved from the MediaWiki page, will appear on the left part of the same page ensuring that users can easily access the annotation information for a gene.

### Editing

New nodes and edges can be added by point-and-click on the main page. Under the editing mode, users can click a blank region to add a new node. A dialog window with a form will appear for users to enter the new gene’s information, including symbol, full name and ID. In order to ensure unique gene IDs and maintain flexibility of editing at the same time, we implemented autosuggestion and autocompletion features for the input form. Thus, users can type in a gene symbol, full name or a Uniprot ID, and a list of possible genes will appear for the user to choose from. In this case, the added gene is automatically assigned a Uniprot ID. Users may manually input new genes without Uniprot IDs via entering a gene ID, symbol, and full name.

A new edge can be added in the same way as a new gene. Users can manually connect two genes under the editing mode. In the dialog window, they will then select the direction (from, to or unknown) and the type (activation or inhibition) of interaction. Different lines represent different types of interactions: solid line with arrow indicates activation; a dashed line with arrow indicates inhibition; and a solid line without arrow indicates other or unknown interactions type (select “unknown” direction). Users can hover over an edge to see the interaction type (“Activates” or “Inhibits”).

MediaWiki (https://www.mediawiki.org/), the open-source software originally developed for Wikipedia, can be easily used to create wiki-like websites. CrowdNodNet has some MediaWiki webpages containing information about the gene network: genes (nodes), gene interactions (edges) and gene annotations. A PHP script parses these webpages and displays the annotation in the main page. Once the MediaWiki pages are modified, the gene annotation shown in the main page is updated accordingly. Therefore, users can edit the gene annotation by modifying MediaWiki pages, much in the same way as editing Wikipedia pages (https://www.mediawiki.org/wiki/Help:Editing_pages). Each gene usually has its own MediaWiki page containing the annotation information. From the main page, the gene’s document appears once a user clicks that gene node. An “edit” link is shown at the top of the document, which allows the user to edit the gene’s MediaWiki page. When a new gene is added and a user wants to annotate that gene, they can create a page using the “Create annotation” link in the gene’s document. Currently, editing is open to everyone and users can edit after registering an account.

### Annotations

A gene’s MediaWiki page is expected to contain the following annotation information: the full gene name, mRNA and protein sequences, mutants, biological functions, gene expression, interactions with other genes, gene evolution, *etc.* A description for each component is listed in Table 1. For example, SYMRK’s UniProt ID is Q8LKX1, and it is mapped to microarray probeset gi21622627_at on the Affymetrix Lotus GeneChip®. Three SYMRK mutant alleles with nonsense mutation or large insertions cause the absence of root hair curling, infection thread and nodule primordia [2]. SYMRK, containing a signal peptide, an extracellular domain, a transmembrane domain and an intracellular protein kinase domain, is required for both nodule and arbuscular mycorrhiza symbiosis. Although one study showed that transcriptional regulation of SYMRK, Nod factor receptor 1 (NFR1) and Nod factor receptor 5 (NFR5) is mutually independent [2], SYMRK is able to form a complex with NFR5 after the extracytoplasmic region is cleaved [33]. A SYMRK-interacting protein 2 (SIP2) was also found to form a protein complex with SYMRK [6]. The SYMRK kinase domain is highly conserved between legumes and actinorhizal plants, but its extracellular regions are highly variable [4]. All the above annotation information for SYMRK is available at http://Crowd.bioops.info/mediawiki/index.php?title=SYMRK. Users can add refernces following the MediaWiki format (https://www.mediawiki.org/wiki/Extension:Cite). Other annotation information can be added, and users are encouraged to annotate the gene in as much detail as possible. Once a user creates a new annotation page, a structured template, including all the necessary annotation sections, is automatically loaded in the editing area so that the user can easily fill in content following detailed guidelines for each section.

**Table 1:** Gene annotation information in CrowdNodNet

Section	Description
mRNA and protein sequences	FASTA sequences or links to UniProt or GenBank. If available, the probeset mapping of Affymetrix Lotus GeneChip® is also shown in this part.
Mutants	Genotypes and phenotypes
Biological functions	The detailed gene function during nodulation and/or arbuscular mycorrhizal (AM) symbiosis
Gene expression	Expression patterns during nodulation and whether expressed in other organs, e.g. root, leaf, and stem.
Interactions with other genes	Experimentally verified gene interactions, e.g. receptor-binding, phosphorylation reactions and protein complexes
Gene evolution	Orthologs in legumes and non-legumes, and their functions in AM, actinorhizal and legume-rhizobial symbiosis

## Conclusions

The online platform, CrowdNodNet, was created for crowdsourcing nodulation gene networks. Researchers can use the platform to interactively visualize and easily edit this, or just about any, gene network. It is expected that this will become a comprehensive and collaborative knowledge base of nodulation-related genes and pathways which should help researchers access integrated information and share new discoveries in legume-rhizobial symbiosis.

The platform allows users to interactively visualize and edit the gene network in a dynamic and interactive manner, and easily access information about the network. Moreover, all the gene annotation information is written on MediaWiki pages. The network can be edited by by point-and-click on the main page and editing gene information is the same as editing a Wikipedia page, so the learning process is relatively short or even negligible. The platform we constructed is focused on a single pathway. As compared with existing pathway and network databases that include all biological processes and many species, the decentralized and single pathway-based database is easy to circulate within a relatively small community. All these features make it easier to attract experts for continued contribution and development.

## Abbreviations

CCAMK, Calcium/calmodulin-dependent kinase; NFR1, Nod factor receptor 1; NFR5, Nod factor receptor 5; NIN, Nodule inception; SIP2, SYMRK-interacting protein 2; SYMRK, Symbiosis receptor-like kinase.
